# Whole genome sequencing of Luxi Black Head sheep for screening selection signatures associated with important traits

**DOI:** 10.5713/ab.21.0533

**Published:** 2022-04-30

**Authors:** Zhaohua Liu, Xiuwen Tan, Jianying Wang, Qing Jin, Xianfeng Meng, Zhongfeng Cai, Xukui Cui, Ke Wang

**Affiliations:** 1 Key Laboratory of Livestock and Poultry Multi-omics of MARA, Institute of Animal Science and Veterinary Medicine, Shandong Academy of Agricultural Sciences, Jinan 250100, China; 2 Shandong Key Laboratory of Animal Disease Control and Breeding, Jinan 250100, China; 3 Heze Animal Husbandry Workstation, Heze 274000, China

**Keywords:** Important Traits, Luxi Black Head Sheep, Selection Signatures, Whole Genome Sequencing

## Abstract

**Objective:**

Luxi Black Head sheep (LBH) is the first crossbreed specialized for meat production and was developed by crossbreeding Black Head Dorper sheep (DP) and Small Tailed Han sheep (STH) in the farming areas of northern China. Research on the genomic variations and selection signatures of LBH caused by continuous artificial selection is of great significance for identifying the genetic mechanisms of important traits of sheep and for the continuous breeding of LBH.

**Methods:**

We explored the genetic relationships of LBH, DP, and several Mongolian sheep breeds by constructing phylogenetic tree, principal component analysis and linkage disequilibrium analysis. In addition, we analysed 29 whole genomes of sheep. The genome-wide selection signatures have been scanned with four methods: heterozygosity (*H*_P_), fixation index (*F*_ST_), cross-population extended haplotype homozygosity (XP-EHH) and the nucleotide diversity (*θ*_π_) ratio.

**Results:**

The genetic relationships analysis showed that LBH appeared to be an independent cluster closer to DP. The candidate signatures of positive selection in sheep genome revealed candidate genes for developmental process (HoxA gene cluster, *BCL2L11*, *TSHR*), immunity (*CXCL6*, *CXCL1*, *SKAP2*, *PTK6*, *MST1R*), growth (*PDGFD*, *FGF18*, *SRF*, *SOCS2*), and reproduction (*BCAS3*, *TRIM24*, *ASTL*, *FNDC3A*). Moreover, two signalling pathways closely related to reproduction, the thyroid hormone signalling pathway and the oxytocin signalling pathway, were detected.

**Conclusion:**

The selective sweep analysis of LBH genome revealed candidate genes and signalling pathways associated with developmental process, immunity, growth, and reproduction. Our findings provide a valuable resource for sheep breeding and insight into the mechanisms of artificial selection.

## INTRODUCTION

**Figure 1:**
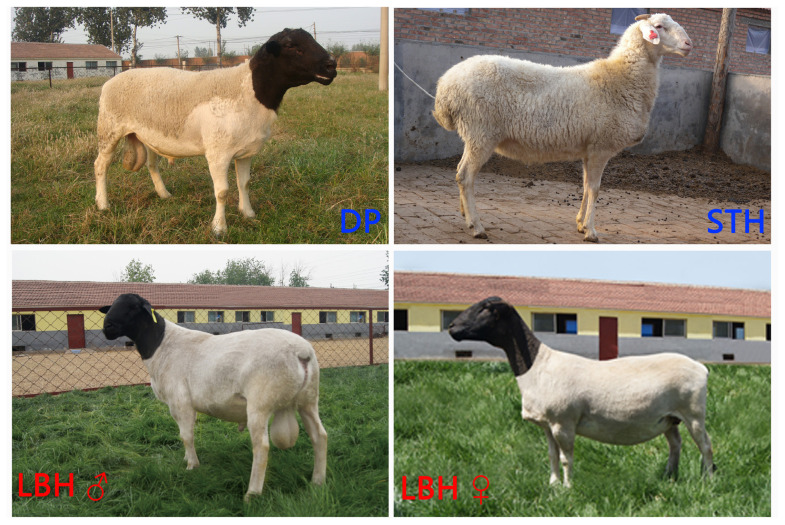
Photos of the pure breeds (DP and STH) and the hybrid (LBH). DP, Black Head Dorper sheep; STH, Small Tailed Han sheep; LBH, Luxi Black Head sheep.

Sheep were initially reared mainly for meat approximately 9,000 years ago and were subsequently specialized for milk and wool approximately 4,000 to 5,000 years ago [1,2]. China has a long history of sheep domestication and rich resources on sheep breeds. Among the modern Chinese sheep breeds, most have a relationship to Mongolian sheep. Small Tailed Han sheep (STH) [3], Hu sheep (H) [4], Tan sheep (T) [5], Wuzhumuqin sheep (WZ) [6] and others are all Mongolian sheep subspecies [3]. In the long process of domestication and selection, they adapted to the environments of various ecological regions and showed outstanding performances, such as the high fecundity of STH and H and the remarkable meat quality of T. However, meat production performance has always been a disadvantage of these indigenous sheep breeds. To meet the increasing consumer demand for mutton, multiple new sheep breeds have been bred after continuous artificial selection and improvement for decades. In the grazing areas of northern China, several developed breeds have been developed and have shown good genetic stability under grazing conditions, such as Bamei mutton sheep [7]. In farming areas, the Luxi Black Head sheep (LBH) breed is the first developed breed specialized for meat production; the LBH breed is a crossbreed of Black Head Dorper sheep (DP) and STH (Figure 1). DP was chosen as the sire because of its stable heritability, fast early growth rate, outstanding meat quality and splendid meat production performance [8]. STH was chosen as dam because it has the advantage of high fecundity, good lactation and motherhood, and can adapt to local forage conditions and ecological environment and have a large stock in Shandong Province [3]. LBH not only has excellent meat production characteristics, but also its average lambing rate is more than 220% [9,10]. Due to the unequivocal target of LBH breeding for more than 20 years, whole-genome selection signature screening of the pure breeds (DP and STH) and the hybrid (LBH) is of great significance for identifying the mechanism of important traits and the sustainable breeding of sheep [11].

With the development of high-throughput sequencing and genotyping technologies, the origin, domestication, and migration of sheep worldwide have been explored [2,12], and the genetic mechanisms of important phenotypic traits of sheep have been gradually revealed and continuously studied, mainly including extreme environmental adaptation mechanisms [13,14], important economic traits such as growth rate [15] and reproduction performance [7] as well as other traits such as tail type [16], horn type [17], ear type [18], coat colour [19], tail fat deposition [20], spine number [6], etc.

According to previous reports, there are abundant methods for investigating animal genome-wide selection signatures. In order to identify favourable alleles and candidate mutations, Rubin et al [21] calculated the pooled heterozygosity (*H*_P_) within each genomic region to detect putative selective sweeps in the chicken genome. In the research of Kijas et al [2], the global fixation index (*F*_ST_) was calculated, which measures differentiation within each sheep breed versus all other breeds and detects both positive and balancing selection. Genomes of the Tibetan horse were scanned for signatures of positive selection using the *F*_ST_ value, the nucleotide diversity (*θ*_π_) ratio and Z-transformed of heterozygosity score (ZH_P_) [22]. To provide insights into rapid genomic adaptations to extreme environments in sheep, four methods, *F*_ST_ value, *θ*_π_ ratio, cross-population extended haplotype homozygosity (XP-EHH) and the program LFMM, were used to test the genome-wide selective sweep [14].

In this study, we undertook genomic analysis of selection signatures with four methods (ZH_P_, Z-transformed of *F*_ST_ [ZF_ST_], XP-EHH, and *θ*_π_ ratio) for 29 whole genomes of sheep (including 10 newly sequenced genomes each for DP and LBH and 9 published genomes for STH) and aimed to identify the genetic basis of the important traits of sheep.

## MATERIALS AND METHODS

### Animal care

The study was conducted according to the guidelines of the Institutional Animal Care and Use Committee of Institute of Animal Science and Veterinary Medicine, Shandong Academy of Agricultural Sciences (IACC20060101).

### DNA samples and sequencing

LBH was bred by crossing DP as sire and STH as dam. Blood samples were collected using the traditional method from 10 LBH from the experimental sheep farm of Shandong Academy of Agricultural Sciences and 10 DP from Qingzhou Haifutong Agricultural Technology Co., Ltd. Genomic DNA was extracted from whole-blood samples using the E.Z.N.A. Tissue DNA kit (Omega Bio-Tek, Norcross, GA, USA). For each sample, at least 3 μg genomic DNA was used for sequencing library construction with an average insert size of 450 bp. The quantified Illumina paired-end library was sequenced in 150-bp paired-end reads on an Illumina HiSeq X. Moreover, 39 publicly available genome sequences from 9 STH and 10 each for H, T, and WZ were employed for comparative analysis.

### Alignments and variant calling

After quality control of the raw sequence reads by Trimmomatic (version 0.36) using the following parameters (LEADING:3, TRAILING:3, SLIDINGWINDOW:4:15, MINLEN:75) [23], all clean reads were mapped to the sheep reference genome (Oar_rambouillet_v1.0) using BWA software for all individuals separately [24]. Polymerase chain reaction duplicates were removed using PICARD tools [25]. Insertion-deletions (INDELs) were realigned using the genome analysis toolkit (GATK) [26]. Single nucleotide polymorphisms (SNPs) and INDELs were called using SAMTools [24]. To exclude SNP calling errors caused by incorrect mapping or INDELs, we did not keep two adjacent SNPs separated by less than 5 bp. To obtain high-quality results for further analyses, we retained only biallelic SNPs and INDELs with calling rates >90% and a quality value >30 (GATK SNP Q30 cut-off). After filtering, the annotation of detected variations was performed by ANNOVAR [27].

### Population genetics analysis

The SNPs were used to calculate the pairwise genetic distances. A neighbour-joining method was used to construct the phylogenetic tree on the basis of the distance matrix, calculated by the software PHYLIP 3.68 [28], and MEGA7 was used to present the phylogenetic tree [29]. Principal component analysis (PCA) was performed according to Patterson et al [30] using EIGENSOFT. To estimate the genome-wide linkage disequilibrium (LD) of each breed, we calculated the mean r^2^ values for pairwise SNPs with PopLDdecay software [31]. Only SNPs with a minor allele frequency >0.05 were used. The parameters of PopLDdecay were set to “-MaxDist 1,000,000 -MAF 0.05 -Het 0.9 -Miss 0.1”.

### Selective sweep analysis

There are many methods to detect selection signatures, but each method has its own limitations. In order to avoid the false positive results, the combination strategy of two or more detection methods is widely used in selection signature detection. Different detection methods could verify each other. Thus, four approaches were used to search the sheep genome for selective sweep regions in this study. First, we calculated the average pooled heterozygosity (*H*_P_) in 100-kb windows with half-step sliding, following the formula described in Rubin et al [21]. Then, we calculated *F*_ST_ values for individual SNPs between LBH and STH as well as LBH and DP and averaged the *F*_ST_ values across each 100-kb window [32]. The distribution of the *H*_P_ and *F*_ST_ values was Z-transformed, and putatively selected windows were extracted at the extreme ends of the distribution by applying Z(*H*_P_) <–4 and Z(*F*_ST_)>4 cut-offs. In addition, we estimated the XP-EHH statistic for STH and DP using LBH as a reference [33]. Furthermore, *θ*_π_ ratios were calculated for LBH versus STH and LBH vs DP, and the *θ*_π_ ratios were log2-transformed. The threshold for identifying selective sweep regions in the XP-EHH and *θ*_π_ ratio tests was set to XP-EHH >0.7 and log_2_(*θ*_π_ ratio) >2. The windows detected by at least two approaches will be considered to have strong selection signatures. Functional genes within or near these windows are considered candidate genes.

### Candidate gene analysis

Ensembl gene annotations were used to identify candidate genes located in the identified selection regions (extending 50 kb up- and downstream). To obtain an accurate understanding of the biological functions and signal pathways of the identified candidate genes, GO functional enrichment analysis was performed using the GOstat program [34], and Kyoto encyclopedia of genes and genomes (KEGG) analysis was performed using the DAVID Bioinformatics Resources [35].

## RESULTS

### Genome resequencing and identification of single nucleotide polymorphisms and INDELs

We generated the genomic sequences of 20 sheep (10 for LBH, 10 for DP) and collected publicly available whole-genome data for an additional 39 sheep (9 for STH and 10 each for T, H, and WZ). In total, 9.1 billion clean reads of 59 sheep samples were aligned to the sheep reference genome (Oar_rambouillet_v1.0). The average alignment rate was 99.56%, and the average depths of the clean reads were 10.38X in the 20 newly sequenced genomes and 5.58X in the 39 publicly available sheep (Supplementary Table S1). After the variant calling and filtering processes, we identified an average of 10.81 million SNPs and 1.40 million INDELs per individual for LBH and DP and 7.68 million SNPs and 0.96 million INDELs per individual for the 39 publicly available sheep (Supplementary Table S2).

### Population structure analysis

**Figure 2:**
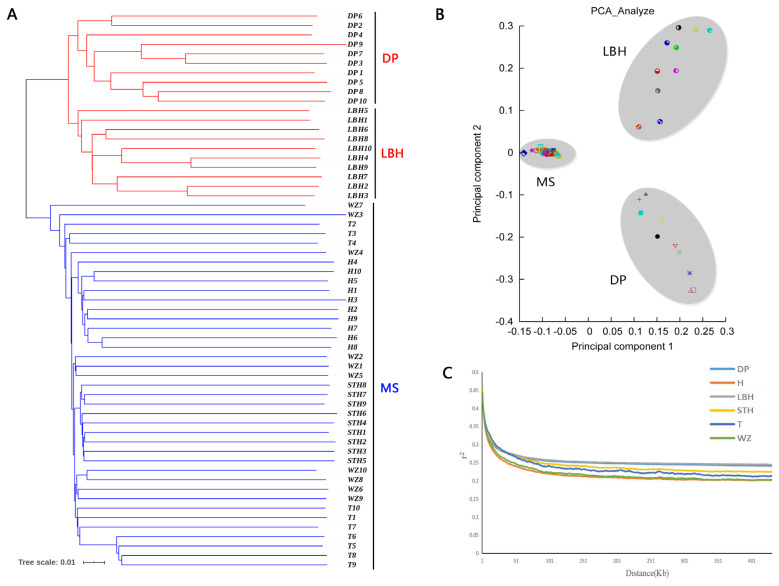
Genetic relationships and linkage disequilibrium decay in six sheep breeds. (a) Phylogenetic tree of 59 individuals. MS is short for Mongolian sheep. (b) Principal component analysis of 59 individuals. (c) Linkage disequilibrium decay in six breeds.

To understand the genetic relationships between LBH and other sheep breeds, we constructed a phylogenetic tree with SNP variants of each individual (Figure 2A), showing that 4 Mongolian sheep breeds (STH, T, H and WZ) gathered into a cluster and LBH and DP gathered together. Furthermore, a PCA of 59 sheep also demonstrated that 4 Mongolian sheep breeds had close genetic relationships; however, LBH and DP belonged to 2 independent clusters (Figure 2B). Analyses of the genome-wide LD pattern indicated that LBH and DP showed an overall slow decay rate and a high level of LD, whereas the Mongolian sheep breeds exhibited a rapid decay rate and a low level of LD. This suggested that LBH and DP have smaller effective population sizes than Mongolian sheep breeds (Figure 2C).

### Detection of selective sweeps

**Figure 3:**
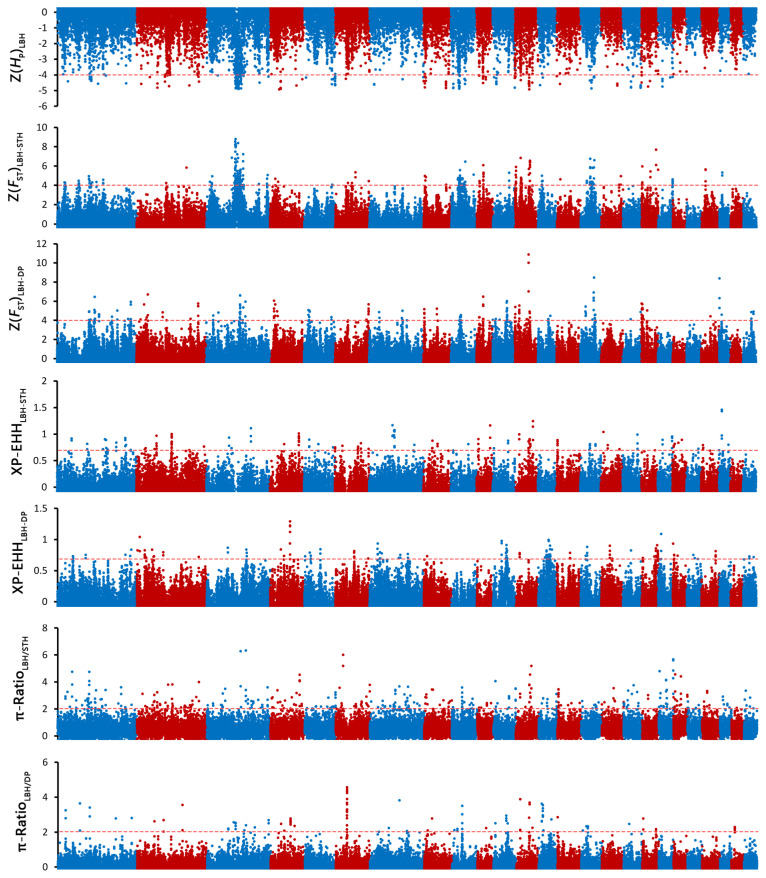
Manhattan plot of genome-wide selection signatures in LBH, DP and STH. ZH_P_, ZF_ST_, XP-EHH and *θ*_π_ ratio values were calculated in 100-kb window with half-step sliding across all autosomes. LBH, Luxi Black Head sheep; DP, Black Head Dorper sheep; STH, Small Tailed Han sheep; ZH_P_ values were calculated for each breed; ZF_ST_ were calculated between LBH and STH as well as LBH and DP; XP-EHH were calculated for STH and DP, using LBH as a reference; *θ*_π_ ratios were calculated for LBH vs STH and LBH vs DP.

**Figure 4:**
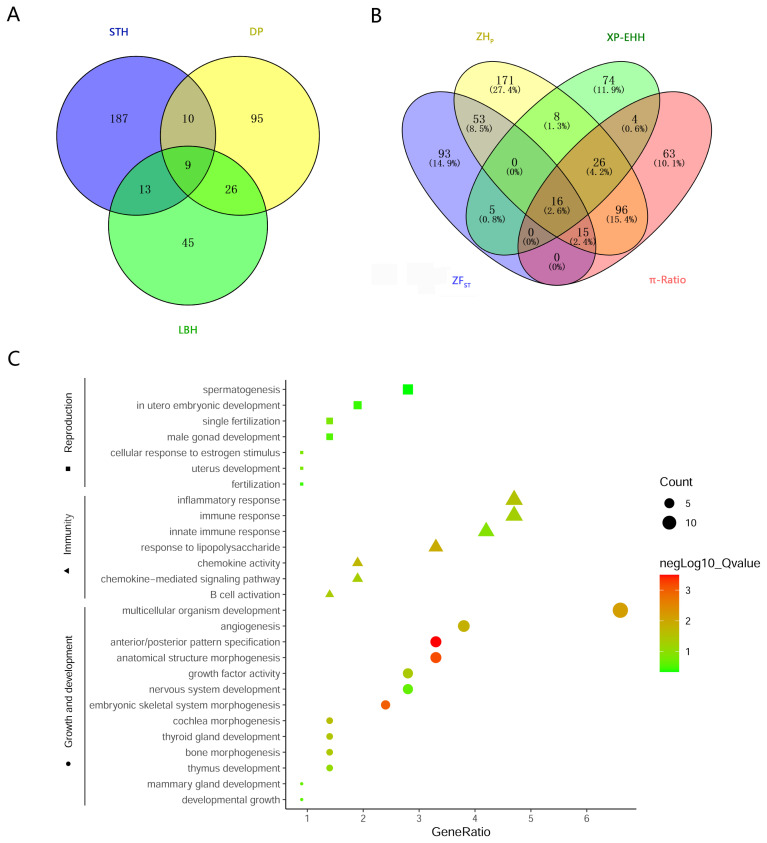
Candidate genes associated with selective sweeps. (a) A Venn plot showing numbers of overlapping candidate genes identified by ZH_P_ test among 3 breeds. (b) A Venn plot showing numbers of overlapping candidate genes identified by the four methods. (c) GO enrichments involved in developmental process, immunity, growth and reproduction for the candidate genes identified by at least 2 tests. ZH_P_, Z-transformed of heterozygosity score; GO, gene ontology.

To investigate the genetic separation between the pure breeds and the hybrid, selective sweep analysis was performed to detect the selection signatures using *H*_P_, *F*_ST_, XP-EHH, and *θ*_π_ ratio tests (Figure 3). First, the *H*_P_ values of LBH, DP and STH were calculated, and 93, 140, and 219 genes (in total 385 genes) were detected with a cut-off of Z(*H*_P_) <–4 from the 3 breeds, respectively (Figure 4A; Supplementary Figure S1; Supplementary Table S3). Among them, 9 genes showed selection signatures in all 3 breeds and were significantly enriched in 11 gene ontology (GO) terms, including positive regulation of cell proliferation, histone deacetylase activity, rhythmic process, etc. (Supplementary Figure S2). In addition, FST, XP-EHH, and θπ ratio tests were performed between LBH and STH as well as LBH and DP. In total, 182 (105 for *F*_ST_ between LBH and STH, 89 for *F*_ST_ between LBH and DP), 133 (50 for XP-EHH_STH to LBH_, 86 for XP-EHH_DP to LBH_) and 220 (164 for *θ*_π__–LBH_/*θ*_π__–STH_, 61 for *θ*_π__-LBH_/*θ*_π__–DP_) genes were detected with cut-offs of Z(*F*_ST_) >4, XP-EHH >0.7, and log_2_(*θ*_π_ ratio) >2, respectively. Among them, 16 genes were detected by all four tests at the same time, and 223 genes were detected by at least two approaches and were considered candidate genes (Figure 4B; Supplementary Table S3). GO analysis revealed that these genes were significantly enriched in 29 GO terms (p<0.05), including anterior/posterior pattern specification, anatomical structure morphogenesis, embryonic skeletal system morphogenesis, etc. Subsequently, we investigated the general functions of candidate genes involved in developmental processes (Homeobox-A (HOXA) cluster genes; B-cell lymphoma 2-like 11 [*BCL2L11*]; thyroid stimulating hormone receptor [*TSHR*]), immunity (C-X-C motif chemokine ligand 6 [*CXCL6*]; *CXCL1*; Src kinase associated phosphoprotein 2 [*SKAP2*]; protein tyrosine kinase 6 [*PTK6*]; macrophage stimulating 1 receptor [*MST1R*]), growth (platelet derived growth factor D [*PDGFD*]; fibroblast growth factor 18 [*FGF18*]; serum response factor [*SRF*]; suppressor of cytokine signalling 2 [*SOCS2*]), and reproduction (breast cancer amplified sequence 3 [*BCAS3*]; tripartite motif containing 24 [*TRIM24*]; astacin like metalloendopeptidase [*ASTL*]; fibronectin type III domain containing 3A [*FNDC3A*]) (Figure 4C). KEGG pathway analysis was also performed. Two signalling pathways closely related to reproduction, the thyroid hormone signalling pathway and the oxytocin signalling pathway, had 6 enriched genes. HRas proto-oncogene (*HRAS*), GTPase and phospholipase C beta 4 (*PLCB4*) were enriched in both pathways. Due to the *H*_P_ and *F*_ST_ tests could not discriminate positive selection or negative selection, the positive selection occurred in the LBH genome has been screened by comparing XP-EHH with them and 120 candidate genes have been detected (Supplementary Table S4). GO analysis revealed that these genes were enriched in regulation of cell growth, in utero embryonic development, spermatogenesis, lactation, xenophagy, viral process, etc.

**Figure 5:**
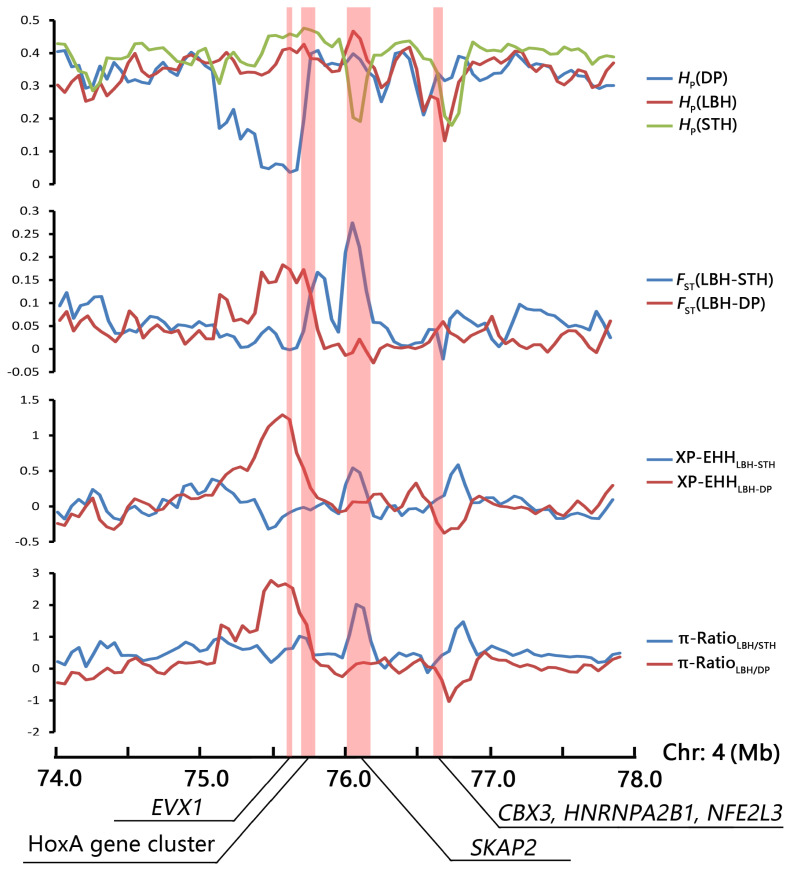
Selection signatures surrounding the HoxA gene cluster region.

A strong selection signature was mapped to the HoxA gene cluster region, including 10 HoxA genes (*HOXA1–7*, *HOXA9*, *HOXA11*, and *HOXA13*). HOX transcription factors play an important regulatory role in embryonic development, cell differentiation, proliferation and apoptosis [36]. In this study, the ten HoxA genes were enriched in several GO terms involved in development (such as anterior/posterior pattern specification, embryonic skeletal system morphogenesis, thyroid gland development) and reproduction (such as single fertilization, uterus development, spermatogenesis). Thus, we investigated the selection pattern of the HoxA gene cluster and adjacent genes in the genomic region of the three sheep breeds (Figure 5). The results indicated that the HoxA gene cluster was under selection only in the genome of DP, whereas another candidate gene, *SKAP2*, related to B cell activation was under selection only in the STH genome.

## DISCUSSION

LBH becomes a new sheep breed through hybridization, transverse cross fixation and generation breeding for more than 20 years, taking excellent meat production performance and high fecundity as the main breeding objectives. Its coat colour is black on the head and neck and white on the body. Both males and females have thin tails and have no horn. The average weight of adult ram is 102.8 kg and that of ewe is 76.8 kg; The ewes are in estrus all year round and could produce three times in two years, with an average lambing rate of more than 220%. The average weight of male lambs is 49.1 kg after intensive fattening from 3 months to 5 months old and the slaughter rate is 56.6% [9,10]. Continuous artificial selection in production-oriented breeding would bring selective signatures to the genome of domesticated sheep [7]. Therefore, this study attempted to identify selection signatures and candidate genes related to economically important traits like the performances of development, immunity, growth, and reproduction by comparing the genome of the pure breeds and the hybrid, aiming to reveal the formation mechanism of these traits and develop new molecular markers for more targeted breeding and continuously improve the meat production and reproductive performance of LBH. Results shown the positive selection of the functional genes in LBH genome that enriched in GO terms “regulation of cell growth” and “in utero embryonic development” might be related to the directional breeding of LBH for meat production. The genes enriched in “spermatogenesis” and “lactation” might relate to the reproduction trait. The genes enriched in “xenophagy” and “viral process” might be involved in the immune system. One of the genes associated with development *SOCS2* is one of the main regulators of growth hormone (GH)/insulin-like growth factor 1 (IGF-1) signalling and has an important role in the regulation of growth and metabolism, which has been reported to be one positively selected gene regulating specific genes in the HIF-1 pathway in a whole-genome study of native sheep [14].

As the sire of LBH, DP is world famous as a meat-producing sheep breed because of its rapid growth. In the present research, we identified that the HoxA gene cluster showed strong signatures of positive selection in the genome of DP and not in LBH or STH (Figure 5). Hox genes are coding for key transcription factors (HOX-TFs), which would pattern the animal body plan. During embryonic development, Hox genes are expressed in overlapping patterns and function in a partially redundant manner [37]. Within the HOX family of homeobox transcription factor genes are the leading candidates for the regulation of embryonic implantation. A crucial role of *HOXA10* in endometrial receptivity has been well established [38], and the highly expressed *HOXA9* and *HOXA11* genes also appear to be involved in endometrial receptivity [39]. In addition, the HoxA gene cluster is critically involved in axial skeleton development, and the expression patterns of these genes are associated with evolutionary changes in the vertebral column [40]. A strong selection signature was detected in the HoxA gene cluster region on the genome of Mongolian sheep, including nine HoxA genes, considered a potential association with vertebral number variations of Mongolian sheep [13].

Adjacent to the HoxA gene cluster, the *SKAP2* gene region showed a selective signature in the genome of STH but not in DP and LBH (Figure 5). As a substrate of Src family kinases, *SKAP2* has been suggested to be a broadly expressed adaptor associated with the control of actin polymerization, cell migration, and oncogenesis [41]. These functions indicate that *SKAP2* plays an important role in the immune system [42] and is a causal candidate gene of some diseases. In addition, *SKAP2* has been reported to be related to the process of oocyte maturation. He et al [43] investigated the expression, localization, and functions of *SKAP2* during mouse oocyte asymmetric division, and the results indicated that *SKAP2* regulates the Arp2/3 complex by interacting with WAVE2 in mouse oocytes, thus affecting the assembly of microfilaments in oocyte meiosis.

Moreover, some candidate genes and signalling pathways previously reported in selection signature research of the sheep genome also appeared in the results of the current study, such as *PDGFD* reported to be a candidate gene for formation of the fat tail of Chinese indigenous sheep and *MC1R* for influencing coat phenotypes [44]. Positive selection surrounding the *TSHR* gene on the sheep genome, which plays a pivotal role in metabolic regulation and the control of reproduction, has already been detected in previous studies [2,3]. The oxytocin signalling pathway involving 6 candidate genes (G protein subunit alpha Q [*GNAQ*]; *HRAS*; calcium/calmodulin dependent protein kinase IV [*CAMK4*]; *CAMKK1*; *PLCB4*; protein kinase AMP-activated catalytic subunit alpha 1 [*PRKAA1*]) was detected in this study, whereas 3 genes (jun proto-oncogene, AP-1 transcription factor subunit [*JUN*]; inositol 1,4,5-trisphosphate receptor type 3 [*ITPR3*]; phospholipase C beta 2 [*PLCB2*]) were involved in this pathway, as detected by genome research in Bamei mutton sheep [7].

In conclusion, in this study, we re-sequenced the whole genomes of 10 LBH individuals, combining with DP and STH and other Chinese domestic sheep to investigate the genetic relationship among them, and to detect the selective signatures in sheep genome. Our results suggested LBH is a new group which is different from the existing breeds and have more prominent meat potential than Chinese local sheep breeds. Several GO terms, signalling pathways, and candidate genes had been to revealed associated with developmental process, immunity, growth, and reproduction. Our findings would provide a better understanding of artificial selection on the sheep genome and be valuable for further sheep breeding.

## SUPPLEMENTARY MATERIAL

Raw sequence data generated in this study have been deposited to the NCBI Sequence Read Archive under the BioProject accession number PRJNA814801.

Supplementary Table S1. Overview of sequencing and alignments statistics

Supplementary Table S2. Number of SNPs and INDELs for each individual

Supplementary Table S3. Gene list for selective sweep results

Supplementary Table S4. List of genes showed positive selection in LBH genome

Supplementary Figure S1. Manhattan plot of genome-wide selective signals in STH and DP identified by ZHP.

Supplementary Figure S2. GO analysis of candidate genes identified by ZHP.

## References

[1] Chessa B, Pereira F, Arnaud F (2009). Revealing the history of sheep domestication using retrovirus integrations. Science.

[2] Kijas JW, Lenstra JA, Hayes B (2012). Genome-wide analysis of the world’s sheep breeds reveals high levels of historic mixture and strong recent selection. PLoS Biol.

[3] Liu Z, Ji Z, Wang G, Chao T, Hou L, Wang J (2016). Genome-wide analysis reveals signatures of selection for important traits in domestic sheep from different ecoregions. BMC Genomics.

[4] Feng X, Li F, Wang F (2018). Genome-wide differential expression profiling of MRNAs and LncRNAs associated with prolificacy in Hu sheep. Biosci Rep.

[5] EEr H, Ma L, Xie X (2020). Genetic polymorphism association analysis of SNPs on the species conservation genes of tan sheep and Hu sheep. Trop Anim Health Prod.

[6] Li S, Luo R, Lai D (2018). Whole-genome resequencing of Ujumqin sheep to investigate the determinants of the multi-vertebral trait. Genome.

[7] Yao Y, Pan Z, Di R (2021). Whole genome sequencing reveals the effects of recent artificial selection on litter size of Bamei Mutton sheep. Animals.

[8] Cloete SW, Snyman MA, Herselman MJ (2000). Productive performance of Dorper sheep. Small Rumin Res.

[9] Cui X, Wang J, Zhang G (2017). Fattening performance test of luxi Blackhead sheep. Shandong Agricultural Sciences.

[10] Cui X, Wang J, Meng X (2018). Study on reproductive performance of Luxi Blackhead sheep. Shandong Agricultural Sciences.

[11] Wang J, Zhao H, Li H, Wang D, Li F (2005). Fattening experiment of F1 crossbreeding between Dorper sheep and Small Tailed Han sheep. Chinese J Anim Sci.

[12] Li X, Yang J, Shen M (2020). Whole-genome resequencing of wild and domestic sheep identifies genes associated with morphological and agronomic traits. Nat Commun.

[13] Wang W, Zhang X, Zhou X (2019). Deep genome resequencing reveals artificial and natural selection for visual deterioration, plateau adaptability and high prolificacy in Chinese Domestic sheep. Front Genet.

[14] Yang J, Li W, Lv F (2016). Whole-genome sequencing of native sheep provides insights into rapid adaptations to extreme environments. Mol Biol Evol.

[15] Pasandideh M, Gholizadeh M, Rahimi-Mianji G (2020). A genome-wide association study revealed five snps affecting 8-month weight in sheep. Anim Genet.

[16] Pan Z, Li S, Liu Q (2019). Rapid evolution of a retro-transposable hotspot of ovine genome underlies the alteration of BMP2 expression and development of fat tails. BMC Genomics.

[17] Pan Z, Li S, Liu Q (2018). Whole-genome sequences of 89 Chinese sheep suggest role of RXFP2 in the development of unique horn phenotype as response to semi-feralization. GigaScience.

[18] Paris JM, Letko A, Häfliger IM, Ammann P, Drögemüller C (2020). Ear type in sheep is associated with the MSRB3 locus. Anim Genet.

[19] Gebreselassie G, Liang B, Berihulay H (2020). Genomic mapping identifies two genetic variants in the MC1R gene for coat colour variation in Chinese Tan sheep. PLoS One.

[20] Xu SS, Ren X, Yang GL (2017). Genome-wide association analysis identifies the genetic basis of fat deposition in the tails of sheep (Ovis Aries). Anim Genet.

[21] Rubin CJ, Zody MC, Eriksson J (2010). Whole-genome resequencing reveals loci under selection during chicken domestication. Nature.

[22] Liu X, Zhang Y, Li Y (2019). EPAS1 gain-of-function mutation contributes to high-altitude adaptation in Tibetan horses. Mol Biol Evol.

[23] Bolger AM, Lohse M, Usadel B (2014). Trimmomatic: A flexible trimmer for Illumina sequence data. Bioinformatics.

[24] Li H, Durbin R (2009). Fast and accurate short read alignment with Burrows-Wheeler transform. Bioinformatics.

[25] PICARD [Internet].

[26] McKenna A, Hanna M, Banks E (2010). The genome analysis toolkit: A MapReduce framework for analyzing next-generation DNA sequencing data. Genome Res.

[27] Wang K, Li M, Hakonarson H (2010). ANNOVAR: functional annotation of genetic variants from high-throughput sequencing data. Nucleic Acids Res.

[28] Felsenstein J (1989). PHYLIP - Phylogeny Inference Package (version 3.2). Cladistics.

[29] Kumar S, Stecher G, Tamura K (2016). MEGA7: Molecular evolutionary genetics analysis version 7.0 for bigger datasets. Mol Biol and Evol.

[30] Patterson N, Price AL, Reich D (2006). Population structure and eigenanalysis. PLoS Genet.

[31] Zhang C, Dong SS, Xu JY, He WM, Yang TL (2019). PopLDdecay: A fast and effective tool for linkage disequilibrium decay analysis based on variant call format files. Bioinformatics.

[32] Weir BS, Hill WG (2002). Estimating F-statistics. Annu Rev Genet.

[33] Sabeti PC, Varilly P, Fry B (2007). Genome-wide detection and characterization of positive selection in human populations. Nature.

[34] Beissbarth T, Speed TP (2004). GOstat: find statistically overrepresented Gene Ontologies within a group of genes. Bioinformatics.

[35] Huang DW, Sherman BT, Lempicki RA (2009). Systematic and integrative analysis of large gene lists using DAVID bioinformatics resources. Nat Protoc.

[36] Mallo M (2018). Reassessing the role of hox genes during vertebrate development and evolution. Trends Genet.

[37] Jerković I, Ibrahim DM, Andrey G (2017). Genome-wide binding of posterior HOXA/D transcription factors reveals subgrouping and association with CTCF. PLoS Genet.

[38] Ashary N, Laheri S, Modi D (2020). Homeobox genes in endometrium: from development to decidualization. Int J Dev Biol.

[39] Xu B, Geerts D, Bu Z (2014). Regulation of endometrial receptivity by the highly expressed HOXA9, HOXA11 and HOXD10 HOX-class homeobox genes. Hum Reprod.

[40] Böhmer C, Werneburg I (2017). Deep time perspective on turtle neck evolution: chasing the hox code by vertebral morphology. Sci Rep.

[41] Ghelman J, Grewing L, Windener F, Albrecht S, Zarbock A, Kuhlmann T (2021). SKAP2 as a new regulator of oligodendroglial migration and myelin sheath formation. Glia.

[42] Dadwal N, Mix C, Reinhold A (2021). The multiple roles of the cytosolic adapter proteins ADAP, SKAP1 and SKAP2 for TCR/CD3-mediated signaling events. Front Immunol.

[43] He S, Xu B, Liu Y (2017). SKAP2 Regulates Arp2/3 complex for actin-mediated asymmetric cytokinesis by interacting with WAVE2 in mouse oocytes. Cell Cycle.

[44] Wei C, Wang H, Liu G (2015). Genome-wide analysis reveals population structure and selection in Chinese indigenous sheep breeds. BMC Genomics.

