# Supplementary material for: Whole genome sequencing of Luxi Black Head sheep for screening selection signatures associated with important traits
Source: Anim Biosci. 2022 Apr 30;35(9):1340–50. doi: 10.5713/ab.21.0533 (PMC9449392; doi:10.5713/ab.21.0533)
Supplement: Supplementary Table S1. — Overview of sequencing and alignments statistics [file ab-21-0533-suppl1.pdf]

Supplementary Table S1. Overview of sequencing and alignments statistics.

Sample	Clean	Mapped-reads	Mapped-rate(%)	Coverage(%)	Aver-dep
H10	135772804	135528412	99.82	94.42	5.83
H1	129932358	129594533	99.74	94.25	5.59
H2	152984610	152632745	99.77	94.84	6.59
H3	147815052	147386388	99.71	94.59	6.36
H4	129531744	129285633	99.81	93.18	5.53
H5	118477370	118252262	99.81	92.29	5.05
H6	148344610	147958914	99.74	94.84	6.39
H7	128584614	128391737	99.85	94.26	5.54
H8	135203312	134986986	99.84	94.41	5.8
H9	141630174	141403565	99.84	92.54	6.04
STH1	124078216	123445417	99.49	93.77	5.29
STH2	139127944	138640996	99.65	94.54	5.99
STH3	133332892	132866226	99.65	94.32	5.74
STH4	132670498	132179617	99.63	94.37	5.72
STH5	134628954	134090438	99.6	94.4	5.81
STH6	130719916	130549980	99.87	92.96	5.61
STH7	117978626	117825253	99.87	93.72	5.08
STH8	130321042	130164656	99.88	94.39	5.61
STH9	115767330	115558948	99.82	93.55	4.97
T10	115346562	115208146	99.88	91.84	4.92
T1	118935034	118697163	99.8	93.53	5.1
T2	142238434	141982404	99.82	93.27	6.08
T3	136273636	136014716	99.81	91.17	5.84
T4	138982098	138704133	99.8	94.28	5.97
T5	108588000	108283953	99.72	92.82	4.63
T6	115261224	114984597	99.76	93.13	4.9
T7	108485502	108225136	99.76	92.64	4.61
T8	113972842	113779088	99.83	91.18	4.85
T9	124425524	124288655	99.89	93.71	5.31
WZ10	116903322	116739657	99.86	93.61	4.97
WZ1	133977808	133642863	99.75	94.13	5.75
WZ2	127985908	127704339	99.78	93.9	5.49
WZ3	124659624	124335508	99.74	93.64	5.36
WZ4	133405320	133098487	99.77	94.26	5.74
WZ5	129710578	129321446	99.7	94.05	5.58
WZ6	126193176	126016505	99.86	94.2	5.44
WZ7	192982370	192615703	99.81	95.42	8.28
WZ8	133414994	133161505	99.81	94.48	5.75

WZ9	109124562	108895400	99.79	86.38	4.65
DP6	173291046	171523477	98.98	94.55	8.82
DP1	231278470	230122077	99.5	95.55	11.82
DP7	258607208	255426339	98.77	95.25	13.12
DP8	232104930	230062406	99.12	95.09	11.8
DP2	187949386	186088687	99.01	94.91	9.55
DP9	206936916	203315519	98.25	91.83	10.43
DP3	159238384	157789314	99.09	93.94	8.1
DP10	210406072	209227797	99.44	95.38	10.75
DP4	175357740	174130235	99.3	94.92	8.95
DP5	171435846	169755774	99.02	94.48	8.68
LBH10	214079544	213201817	99.59	95.43	10.91
LBH1	175552884	174587343	99.45	95.02	8.98
LBH2	286460062	284741301	99.4	95.73	14.58
LBH3	222404558	221203573	99.46	95.3	11.34
LBH4	200857630	199190511	99.17	94.84	10.2
LBH5	223126506	221408431	99.23	95.31	11.35
LBH6	194072106	193101745	99.5	94.62	9.9
LBH7	188436604	187550951	99.53	95.02	9.62
LBH8	197549280	192788342	97.59	95.17	9.88
LBH9	174765430	173524595	99.29	94.99	8.91
