# Supplementary material for: Whole genome sequencing of Luxi Black Head sheep for screening selection signatures associated with important traits
Source: Anim Biosci. 2022 Apr 30;35(9):1340–50. doi: 10.5713/ab.21.0533 (PMC9449392; doi:10.5713/ab.21.0533)
Supplement: Supplementary Table S2. — Number of SNPs and INDELs for each individual [file ab-21-0533-suppl2.pdf]

Supplementary Table S2. Number of SNPs and INDELs for each individual.

type	all-snp	hom	het	all-indel	deletion	insertion
DP6	9672059	3201452	6470607	1241919	663612	578307
DP1	12438021	4292373	8145648	1614804	864828	749976
DP7	11670610	4262568	7408042	1570670	841038	729632
DP8	11219186	4182409	7036777	1486053	793821	692232
DP2	10619055	3486889	7132166	1357282	725618	631664
DP9	8660367	3657903	5002464	1144036	608718	535318
DP3	8987985	2970764	6017221	1154429	617411	537018
DP10	11688608	4084076	7604532	1514689	809945	704744
DP4	10637831	3364648	7273183	1342507	718336	624171
DP5	9577390	3296065	6281325	1218973	652347	566626
LBH1	10735173	3501354	7233819	1340471	716144	624327
LBH10	11768829	4072743	7696086	1526248	816281	709967
LBH2	12756644	4740920	8015724	1696605	908985	787620
LBH3	11810406	4128277	7682129	1538961	823950	715011
LBH4	10647100	3796888	6850212	1375886	736535	639351
LBH5	11839420	4082720	7756700	1524363	815712	708651
LBH6	9860029	3424028	6436001	1268580	678821	589759
LBH7	10601813	3705142	6896671	1358692	726378	632314
LBH8	10741591	3893514	6848077	1384017	739206	644811
LBH9	10344178	3398417	6945761	1307740	698422	609318
STH1	7178855	1192046	5986809	814087	437711	376376
STH2	8451491	1643455	6808036	958274	512633	445641
STH3	8042255	1511775	6530480	909375	486986	422389
STH4	8073289	1526010	6547279	917762	491283	426479
STH5	8247280	1567067	6680213	934321	499811	434510
STH6	7672501	1520274	6152227	851883	456879	395004
STH7	6800699	1077478	5723221	775766	416742	359024
STH8	7866754	1278597	6588157	895908	480140	415768
STH9	6514450	971283	5543167	755980	406826	349154
T1	7189604	1209562	5980042	822464	441102	381362
T10	6742922	1089959	5652963	747062	402594	344468
T2	8563845	1847267	6716578	927151	497105	430046
T3	8108020	1815307	6292713	872238	469316	402922
T4	8555267	1680050	6875217	947285	506937	440348
T5	6295073	854365	5440708	742510	399461	343049
T6	6755489	1018096	5737393	765812	413335	352477
T7	6166687	831456	5335231	706489	381528	324961
T8	6559603	1003700	5555903	724620	391524	333096

T9	7344865	1212630	6132235	827717	445455	382262
WZ1	8080121	1514347	6565774	904591	484029	420562
WZ10	6154441	760649	5393792	743073	399022	344051
WZ2	7680256	1345542	6334714	867950	465197	402753
WZ3	6104619	1621297	4483322	693448	369585	323863
WZ4	8149840	1481759	6668081	922277	492474	429803
WZ5	7836614	1406107	6430507	888706	475395	413311
WZ6	7636596	1235017	6401579	873974	466956	407018
WZ7	10931301	2960428	7970873	1252508	663523	588985
WZ8	8063945	1370109	6693836	925716	495185	430531
WZ9	6422900	1057338	5365562	681913	368334	313579
H1	7886245	1416083	6470162	896807	480033	416774
H10	7767571	1434094	6333477	895518	479119	416399
H2	9396336	2103563	7292773	1056253	564542	491711
H3	8664932	2018643	6646289	972132	519027	453105
H4	7862988	1376543	6486445	853590	458920	394670
H5	6835376	1154004	5681372	746525	402623	343902
H6	8999583	1904080	7095503	1012308	540645	471663
H7	7724793	1351805	6372988	875308	469210	406098
H8	7839462	1377290	6462172	912524	487934	424590
H9	8277630	1809880	6467750	885712	476580	409132
