# Supplementary material for: Whole genome sequencing of Luxi Black Head sheep for screening selection signatures associated with important traits
Source: Anim Biosci. 2022 Apr 30;35(9):1340–50. doi: 10.5713/ab.21.0533 (PMC9449392; doi:10.5713/ab.21.0533)
Supplement: Supplementary Table S3. — Gene list for selective sweep results [file ab-21-0533-suppl3.pdf]

Supplementary Table S3. Gene list for selective sweep results.

type	count	gene list
Z(H_P)$_{LBH}$ < -4	93	DCAF6, MAB21L1, HSPH1, PIBF1, KLF5, KATNAL1, FLT1, LMO7, NAA38, KAT7, DLX4, Metazoa_SRP, CARD14, SLC26A11, RNF213, U6, ENDOV, CALCOCO2, ATP5MC1, UBE2Z, TRAPPC1, KCNAB3, RNF227, KCNT2, ARL8A, LMOD1, RNF24, C20orf194, DDRGK1, LZTS3, FASTKD5, UBOX5, AVP, ZNF341, PLXDC2, PLCB4, CENPN, BCO1, C6, HCAR1, RSRC2, ZCCHC8, TECPR2, RCOR1, TRAF3, SNUPN, PTPN9, SIN3A, MAN2C1, RETREG2, SPAG16, U2, GLI2, APOBEC2, NFYA, MCUR1, SNRNP200, TMEM127, STARD7, DUSP2, ASTL, ADRA2B, ACOXL, BCL2L11, MERTK, TMEM87B, FBLN7, ZC3H6, GLIPR1, ZDHHC17, NFU1, GFPT1, ACSS3, MRPL42, SOCS2, CRADD, MGAT4A, COA5, TMTC2, ACO2, CSDC2, MEI1, SEMA3D, DNAH11, KRBA1, Bst-2B, BST-2A, CCDC194, PPIP5K2, MACIR, MAN2A1, CAMK4, FAM13A
Z(H_P)$_{DP}$ < -4	140	IGF2BP2, UHMK1, UAP1, LIPH, CPT2, MAB21L1, FNDC3A, MLNR, CDADC1, NAA38, TRAPPC1, KCNAB3, RNF227, UBE2J2, C1QTNF12, SDF4, TNFRSF4, TNFRSF18, C1orf159, RNF223, PAPPA2, RERE, DEFB115, C20orf194, DHTKD1, DDRGK1, LZTS3, FASTKD5, UBOX5, AVP, SEC61A2, CDC123, RGS19, SOX18, U6, SAMD10, ZNF512B, UCKL1, ZBTB46, ZNF276, CENPN, TCF25, MC1R, TUBB3, DEF8, BCO1, GAS8, GPT2, KLHL36, USP10, CRISPLD2, ZFPM1, ZC3H18, IL17C, CTU2, SPG7, RPL13, CPNE7, DPEP1, CDK10, OR4C16, DTX4, SMIM15, RPL37, PRKAA1, TTC33, SLC38A9, PLPP1, DHX29, HCAR1, RCOR1, TRAF3, LBHD2, TNFAIP2, TM6SF1, SNUPN, PTPN9, SIN3A, MAN2C1, VOPP1, MON1A, MST1R, CAMKV, TRAIP, 5S_rRNA, HIBCH, KDM4C, SPAG16, SLC22A23, TUBB2A, TUBB2B, APOBEC2, NFYA, MERTK, TMEM87B, FBLN7, ZC3H6, ZDHHC17, PYM1, SARNP, CD63, TIA1, C2orf42, THADA, MRPL42, SOCS2, CRADD, LYG2, MITD1, TSGA10, GTSF1, SMAGP, POU6F1, TFCP2, CSRNP2, ZC3H7B, TEF, ACO2, CSDC2, MEI1, CCDC134, HOXA13, HOXA11, HOXA9, HOXA7, HOXA6, HOXA5, HOXA4, HOXA3, HOXA2, HOXA1, HDAC9, ARHGAP26, NCAPG, SPRED1, SLC7A7, C14orf93, AJUBA, PRMT5, RBM23
Z(H_P)$_{STH}$ < -4	219	FCGR2B, FCRLA, FCRLB, LURAP1, RAD54L, DUSP12, ATF6, UQCRH, NSUN4, FAAH, HAO2, HSD3B1, RNPC3, EPHX3, NOTCH3, PFN2, ANKUB1, COMMD2, TIPRL, SFT2D2,

		FCGR1A, HIST2H2AC, BOLA1, COL6A2, LHX8, MTMR6, AMER2, FLT1, TOP2A, NAA38, BCAS3, ZBTB4, ZZEF1, PLSCR3, SLC26A11, ATP2A3, RNF213, P2RX1, CAMKK1, NCBP3, NEURL4, GPS2, YBX2, CLDN7, CTDNEP1, GABARAP, NPEPPS, U6, KPNB1, TBKBP1, TBX21, ENDOV, TRAPPC1, KCNAB3, RNF227, TEX50, KLHL20, DARS2, ARL8A, LMOD1, CSRP1, PHLDA3, TNNI1, STMN3, GMEB2, SRMS, ADRA1D, PTK6, RNF24, EEF1A2, KCNQ2, CHRNA4, YTHDF1, DHTKD1, SEC61A2, CDC123, UCKL1, ZBTB46, ARFRP1, OTUD1, PLCG2, KIAA0513, PDGFD, SCUBE2, MMP7, NRIP3, U3, TMEM9B, ASCL3, C11orf16, DENND2B, PLCXD3, C6, U2, DAB2, PLPP1, DHX29, FGF18, COMT, HCAR1, RSRC2, ZCCHC8, TPCN1, ADAL, TUBGCP4, MAP1A, DGLUCY, PTPN9, SIN3A, MAN2C1, RPP25, COX5A, FAM219B, LTF, LHFPL4, PAX5, GNAQ, BMPR2, PNKD, CNTNAP5, CATIP, SLC11A1, CTDSP1, VIL1, CNOT9, PLCD4, BCS1L, MAP2, MARCKSL1, TMEM234, NOP58, GMDS, BICRAL, PTCRA, PTK7, CNPY3, GNMT, PPP2R5D, KLHDC3, RRP36, KLC4, SRF, CUL9, ABCC10, TJAP1, POLR1C, POLH, DEAF1, SCT, CDHR5, MS4A8, IRF7, LMNTD2, HRAS, RNH1, DLG2, SIGIRR, PAPSS2, BTBD16, PLEKHA1, HTRA1, SNN, PDGFA, BFAR, RRN3, NDE1, EFEMP1, NCF4, MPST, FEZ2, LGALS1, NOL12, H1F0, GCAT, GALR3, THADA, TMEM131, TUBA8, CDC42EP1, GGA1, PDXP, SKAP2, SEMA3D, AKR1D1, TRIM24, HDAC9, U7, ASB15, GABRA1, GABRG2, PCDHB14, PCDHB15, PCDHGC3, PCDHGB5, ARHGAP26, WDR36, CAMK4, CXCL8, CXCL6, PPBP, CXCL1, EPGN, CPLX1, SLC49A3, ATP5ME, TMEM271, H2AZ1, DNAJB14, LAMTOR3, PDGFRA, NOP14, KLF3, ATP8B4, TSHR, ALDH1A2, ARID1B, FBXL4, CALB1, HAS2
$Z(F_{ST})_{LBH\text{-}STH} > 4$	105	FCGR2B, FCRLA, FCRLB, DUSP12, ATF6, KRTAP11-1, KAP7, KAP8, KRTAP8-2, U6, PLPPR4, VCAM1, DCAF6, RNF11, HSPH1, UBL3, SLC7A1, SLC46A3, LNX2, MTIF3, UBAC2, Metazoa_SRP, CARD14, SLC26A11, RNF213, NPEPPS, KPNB1, TBKBP1, TBX21, LRRC46, SP2, PAPPA2, STMN3, GMEB2, SRMS, PTK6, EEF1A2, KCNQ2, CHRNA4, YTHDF1, NPBWR2, LKAAEAR1, RGS19, SOX18, UCKL1, ZBTB46, ARFRP1, PLXDC2, MLLT10, PLCB4, KIAA1217, ANKEF1, SNAP25, ZFPM1, ZC3H18, PLCXD3, C6, RPL37, PRKAA1, TTC33, U2, DAB2, CRYBB3, CRYBB2, GRK3, MED13L, CFAP100, CMC1, LRRC2, LTF, MSRA, GMDS, SNN, SNRNP200, TMEM127, STARD7, DUSP2, ASTL, ADRA2B, GPAT2, ACOXL, BCL2L11, TRHDE, HPCAL1, STRBP, MRPL42, SOCS2, CRADD, MGAT4A, COA5, METAP2,

		TMEM131, TMTC2, KANSL3, NEURL3, FAM83F, TNRC6B, TUBA8, CDC42EP1, GGA1, PDXP, HDAC9, KIT, KLHL5, WDR19
$Z(F_{ST})_{LBH-DP}$ > 4	89	RGS4, DCAF6, LATS2, XPO4, EEF1AKMT1, LNX2, MTIF3, RNF213, U6, ENDOV, RPTOR, PRELP, OPTC, ATP2B4, CFAP74, GNB1, NADK, SLC35E2B, UBE2J2, C1QTNF12, SDF4, TNFRSF4, TNFRSF18, C1orf159, RNF223, HES4, NOC2L, ZBTB17, TCFL5, DHTKD1, SEC61A2, CDC123, TAF4, CAMK1D, NPBWR2, LKAAEAR1, RGS19, SOX18, PLCB1, PLCB4, ERCC8, ELOVL7, PDE4D, RCOR1, TRAF3, RBMS3, TGFBR2, PNKD, CATIP, SLC11A1, CTDSP1, VIL1, 5S_rRNA, UBAP2, NOL6, AQP3, RUFY4, KCTD1, NUBP1, TELO2, TMEM204, CRAMP1, JPT2, MAPK8IP3, EME2, NUBP2, FAHD1, MAK16, GOLGA7, GINS4, GPAT4, SENP1, COL2A1, HDAC7, RAPGEF3, ENDOU, RPAP3, METAP2, HDAC9, IL5, IRF1, RFX2, RANBP3, NDUFA11, KIF3A, SEPTIN8, NOP14, TNIP2, TRDN
$XP\text{-}EHH_{STH\ to\ LBH}$ > 0.7	50	PFN2, LHX8, CD1D, SLC16A5, ARMC7, NUP85, MRPS7, BCAS3, TEX50, KLHL20, STMN3, GMEB2, SRMS, PTK6, EEF1A2, KCNQ2, DHTKD1, SEC61A2, RPN2, MANBAL, SRC, ZBTB46, ARFRP1, U2, DGLUCY, GMDS, BICRAL, PTCRA, PTK7, CNPY3, GNMT, PPP2R5D, KLHDC3, RRP36, KLC4, RAB38, SNN, KCNG3, COX7A2L, AKR1D1, TRIM24, PCDHGC3, PCDHGB5, CXCL8, CXCL6, PPBP, TSHR, LIN52, VSX2, VRTN
$XP\text{-}EHH_{DP\ to\ LBH}$ > 0.7	86	P2RY12, P2RY13, GPR87, PIP5K1A, PSMD4, ZNF687, NEXN, DNAJB4, GIPC2, U6, AHCTF1, DHTKD1, SEC61A2, CDC123, WWP2, LRP3, CEBPG, AP1G1, PHLPP2, TAT, ZNF507, DPY19L3, CES5A, ERCC8, ELOVL7, IL6ST, SLC38A9, SCAP, KIF9, SETD2, GATA2, DNAJB8, RUVBL1, MGLL, TPRA1, U2, CELSR3, SLC26A6, TMEM89, UQCRC1, RBMS3, UCN2, PFKFB4, CCDC51, PLXNB1, CDC25A, SMARCC1, CSPG5, ELP6, BICD2, IPPK, ECM2, ASPN, OMD, GOLM1, TRAF1, PHF19, PSMD5, FBXW2, 5S_rRNA, STK36, TTLL4, CYP27A1, WNT6, SLC46A2, INIP, ARMC12, CLPSL2, LHFPL5, DPCD, SLC38A1, NELL2, HOXA13, HOXA11, HOXA9, HOXA7, HOXA6, HOXA5, HOXA4, HOXA3, HOXA2, HOXA1, CDHR3, IL5, IRF1, STK32A
$\log_2(\theta_{\pi-LBH}/\theta_{\pi-STH})$ > 2	164	FCGR2B, FCRLA, FCRLB, DUSP12, ATF6, CPNE4, CADM2, FCGR1A, HIST2H2AC, BOLA1, LHX8, GPC5, GPC6, TMEM104, OTOP3, CDR2L, MRPL58, KCTD2, SLC16A5, ARMC7, NUP85, MRPS7, BCAS3, ZBTB4, TEX2, PLSCR3, NEURL4, GPS2, YBX2, CLDN7, CTDNEP1, GABARAP, PHF23, DVL2, DLG4, ASGR2, TEX50, KLHL20, DARS2, ARL8A,

		LMOD1, CSRP1, PHLDA3, TNNI1, STMN3, GMEB2, SRMS, PTK6, EEF1A2, KCNQ2, CHRNA4, DHTKD1, SEC61A2, CDC123, ZBTB46, ARFRP1, OSCAR, YBX1, U6, KIAA0513, OR51E2, PDGFD, FGF18, COMT, RANBP1, ZDHHC8, GASK1B, ADAL, TUBGCP4, NGRN, DGLUCY, RPP25, COX5A, FAM219B, CCL21, LRP1B, PNKD, CNTNAP5, CATIP, SLC11A1, CTDSP1, VIL1, CNOT9, NMRK1, GMDS, U3, TRIM26, BICRAL, PTCRA, PTK7, CNPY3, GNMT, PPP2R5D, OR2B3, KLHDC3, RRP36, KLC4, SRF, CUL9, COL21A1, DYA, IGF2, PRR33, DEAF1, CDHR5, IRF7, LMNTD2, HRAS, RNH1, DLG2, SIGIRR, GRK5, NOL4, SNN, NDE1, CTF1, FBXL19, LGALS1, NOL12, H1F0, GCAT, GALR3, EIF3L, MICALL1, POLR2F, PICK1, TUBA8, CDC42EP1, GGA1, PDXP, SKAP2, AMPH, TRIM24, ABCB1, ATG10, WDR36, CAMK4, SPINK6, SLC4A4, CXCL8, CXCL6, PPBP, CXCL1, EPGN, FRAS1, TMEM271, H2AZ1, DNAJB14, LAMTOR3, CAMK2D, PDGFRA, TBCK, SLIRP, ADCK1, TSHR, TTLL5, DNAAF4, TRAPPC3L, LAMA2, CD24, CYCS, TRIQK, U2, HAS2
$\log_2(\theta_{\pi\text{–LBH}}/\theta_{\pi\text{–DP}}) > 2$	61	IGF2BP2, UHMK1, UAP1, LIPH, CPT2, FNDC3A, MLNR, CDADC1, RERE, DEFB115, DHTKD1, SEC61A2, CDC123, ZNF276, TCF25, MC1R, TUBB3, DEF8, GAS8, KLHL36, USP10, CRISPLD2, DPEP1, CDK10, OR4C16, SMIM15, PLPP1, DHX29, TM6SF1, U6, MON1A, MST1R, CAMKV, TRAIP, UBA7, CDHR4, IP6K1, GMPPB, AMIGO3, MST1, 5S_rRNA, ZNF33B, LYG2, MITD1, TSGA10, GTSF1, ZC3H7B, TEF, ACO2, CSDC2, HOXA13, HOXA11, HOXA9, HOXA7, HOXA6, HOXA5, HOXA4, NCAPG, SPRED1, PDSS2, U2
