# Supplementary material for: Whole genome sequencing of Luxi Black Head sheep for screening selection signatures associated with important traits
Source: Anim Biosci. 2022 Apr 30;35(9):1340–50. doi: 10.5713/ab.21.0533 (PMC9449392; doi:10.5713/ab.21.0533)
Supplement: Supplementary Table S4. — List of genes showed positive selection in LBH genome [file ab-21-0533-suppl4.pdf]

Supplementary Table S4. List of genes showed positive selection in LBH genome.

count	gene list
120	U6, Metazoa_SRP, HSPH1, CARD14, SLC26A11, RNF213, ENDOV, PLXDC2, PLCB4, U2, TMTC2, C6, DCAF6, TRAF3, ASTL, ADRA2B, ACOXL, MGAT4A, COA5, STARD7, DUSP2, BCL2L11, MRPL42, SOCS2, CRADD, SNRNP200, TMEM127, KATNAL1, KAT7, ATP5MC1, UBE2Z, ACSS3, MAN2A1, CALCOCO2, KRBA1, PIBF1, KLF5, LMO7, DLX4, PPIP5K2, MACIR, HCAR1, RETREG2, CENPN, BCO1, MERTK, TMEM87B, FBLN7, ZC3H6, GLIPR1, NFU1, GFPT1, ACO2, CSDC2, MEI1, DNAH11, FAM13A, MAB21L1, UBL3, SLC7A1, NPEPPS, KPNB1, TBKBP1, TBX21, LRRC46, SP2, RPTOR, PLCB1, ANKEF1, SNAP25, CRYBB3, CRYBB2, GRK3, MSRA, 5S_rRNA, GOLGA7, GINS4, GPAT4, SLC46A3, MLLT10, KIAA1217, DAB2, CDC42EP1, GGA1, PDXP, TCFL5, TAF4, JPT2, MAPK8IP3, EME2, NUBP2, FAHD1, TRDN, KRTAP11-1, KAP7, KAP8, KRTAP8-2, RBMS3, TRHDE, PAPPA2, RPL37, PRKAA1, TTC33, GPAT2, TMEM131, KLHL5, WDR19, PLPPR4, HPCAL1, KANSL3, ZFPM1, SENP1, COL2A1, STRBP, CAMK1D, METAP2, NEURL3, TNRC6B, HDAC9, ZC3H18
