# Supplementary material for: Whole genome sequencing of Luxi Black Head sheep for screening selection signatures associated with important traits
Source: Anim Biosci. 2022 Apr 30;35(9):1340–50. doi: 10.5713/ab.21.0533 (PMC9449392; doi:10.5713/ab.21.0533)

Supplementary Figure S1. Manhattan plot of genome-wide selective signals in STH and DP identified by ZH$_P$.

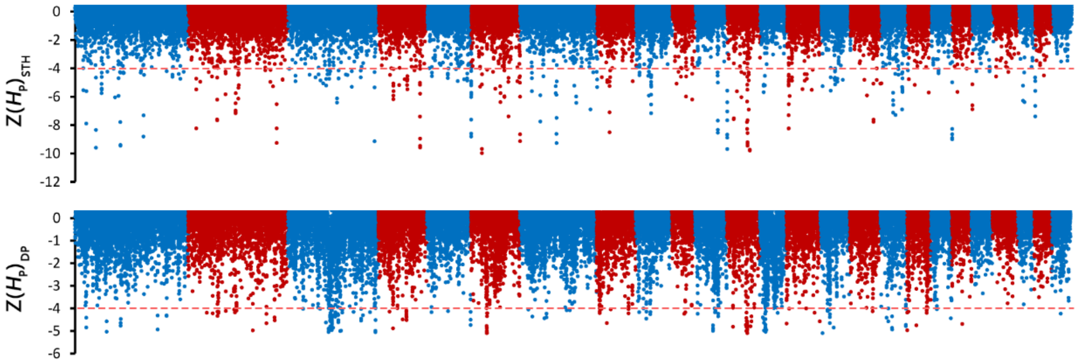

Supplement: Supplementary Figure S1. — Manhattan plot of genome-wide selective signals in STH and DP identified by ZHP. [file ab-21-0533-suppl5.pdf]
