# Supplementary figures and images for: Whole genome sequencing of Luxi Black Head sheep for screening selection signatures associated with important traits
Source: Anim Biosci. 2022 Apr 30;35(9):1340–50. doi: 10.5713/ab.21.0533 (PMC9449392; doi:10.5713/ab.21.0533)

**Supplementary Figure S2.** GO analysis of candidate genes identified by ZHp.

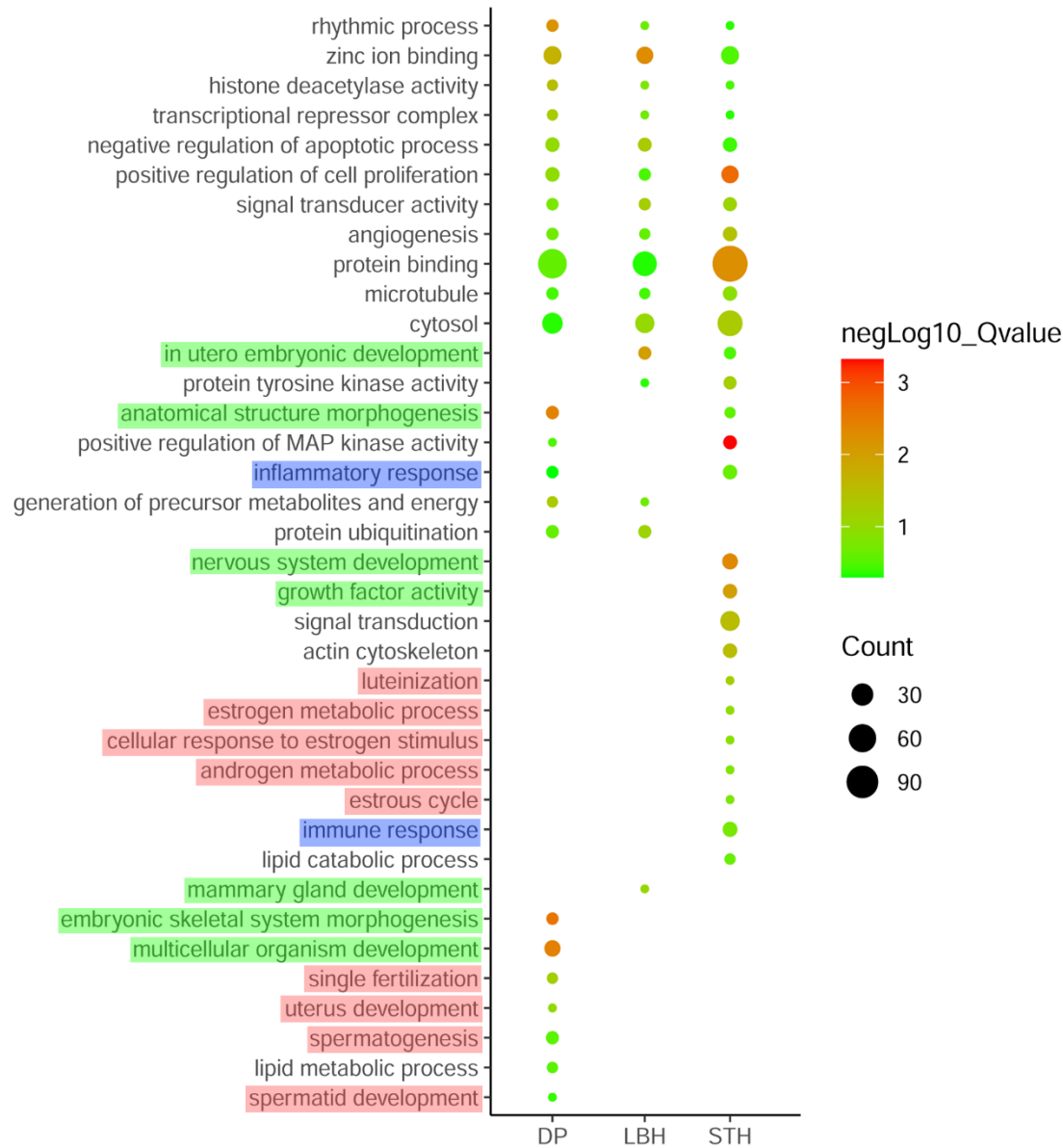

Supplement: Supplementary Figure S2. — GO analysis of candidate genes identified by ZHP. [file ab-21-0533-suppl6.pdf]
